# Exploring University Students’ Perspectives on Animal Experimentation: Insights From a Cross-Sectional Study

**DOI:** 10.7759/cureus.63904

**Published:** 2024-07-05

**Authors:** Ahmed N Canatan, Gizem Çakır, Fatemeh Daneshgar, Ege Pastırmacıoğlu, Söğüt Yorgancı, Berfu Ç Öngün

**Affiliations:** 1 General Practice, School of Medicine, Marmara University, Istanbul, TUR; 2 Anatomy, Faculty of Medicine, Eastern Mediterranean University, Famagusta, CYP

**Keywords:** animal welfare, attitudes and beliefs, animal rights, animal research, animal ethics, animal experimentation

## Abstract

Introduction

Attitudes toward animal experimentation are rapidly evolving with time. This cross-sectional study intends to assess the attitudes of university students at Eastern Mediterranean University toward animal research based on different factors and lifestyle choices.

Materials and methods

Stratified random sampling was used. A total of 215 participants were chosen from the Faculties of Medicine, Pharmacy, Law, and the Department of Psychology (Faculty of Arts and Sciences). An open-access, self-administered, 14-question questionnaire was used. Mann-Whitney U tests were used for score comparisons.

Results

The response rate was 213 (99.07%). Eighty-eight (41.31%) of the participants were male. The mean age was 21.72 ± 2.23. Mann-Whitney U tests revealed significant differences for Q4, Q10, and Q14 (p = 0.012, 0.020, and 0.016, respectively) with respect to gender. Being a pet owner significantly affected the mean scores of Q7 and Q10 (p = 0.046 and p = 0.000, respectively).

Conclusion

The present study reveals nuanced student attitudes toward animal experimentation, balancing concern for animal welfare with the necessity of research. Gender and pet ownership significantly influence these views. The findings underscore the need for continued education on humane and ethical research practices. Future studies should broaden the demographic scope to better understand and track these attitudes over time.

## Introduction

It has become clear that a number of people are very passionate with regard to the ethics and suitability of using animals in experiments [1]. Some might expect that those involved in animal experimentation would have different opinions on the appropriateness of such research than those outside the lab [1]. In truth, it was found that those in support of animal rights were more opposed to the use of animals in research in comparison to scientists or laypersons [1]. However, deeper investigation revealed that scientists were only supportive of the use of animals in the laboratory for research and disinterested in their use for entertainment or decoration purposes [1]. The type of animal experimented on, as well as the severity of the illnesses studied, were also found to be indicators of attitudes toward animal experimentation [2]. Individuals with a background in university education involving animal research were more accepting of the use of animals in the laboratory setting [2]. Furthermore, it was found that the majority of pet owners do not find it acceptable to use pet species in biomedical research [3]. The field of study of a student was found to be a predictor of attitudes toward animal experimentation [3]. Thought-provokingly, females were found to be more opposed to animal research than their male counterparts [4]. Unsurprisingly, vegetarianism was linked with a lower acceptance of the use of animals in research [5]. It is interesting to note that, although some may be uneasy regarding the well-being of the animals, the same individuals also understand the benefit of such research and are more understanding of its necessity [4]. All things considered, it seems that multiple factors influence an individual’s attitudes toward the use of animals in research and that the debate is more complicated than initially thought.

In 1959, the 3R principle was defined in a book titled “The Principles of Humane Experimental Technique” by Russell and Burch [6]. The 3R principle laid the foundations for the implementation of more humane animal research and has since been adapted and reformulated by many countries to be more suitable for modern experimental practice. 3R stands for replace, reduce, and refine. If an experiment can be done without the use of animals, then the alternative model should be used [6]. Only the minimum number of animals required to achieve reliable results should be used, and no more [6]. Finally, the pain and suffering of animals used in research should be reduced as much as possible [6]. The 3R principles do not intend to eliminate animal experimentation; rather, they aim to enhance animal well-being and scientific quality where the use of animals cannot be evaded [6]. Although the 3Rs are internationally accepted, more recognition must be given to their importance, especially by educating those directly involved with using animals in research and those responsible for approving their use in the laboratory environment [7].

Throughout the years, there has been an increase in the number of animal experiments as well as awareness of animal rights [8]. This study aims to assess the attitudes of university students toward animal experimentation and how different factors (pet ownership, vegetarianism, hobbies, field of study, etc.) may influence their perceptions. By focusing on students from Eastern Mediterranean University (EMU), we aim to provide a snapshot of contemporary attitudes that can be reflective of broader changes in societal views toward animal experimentation. This university was selected due to its diverse range of faculties that encompass both medical and nonmedical disciplines, offering a comprehensive perspective on how educational background influences these attitudes. The study also aims to gauge how attitudes toward animal research change over time; the results of our study will be compared to those of two other studies, one from 1988 and the other from 2014, both of which used the same questionnaire [4,9]. This longitudinal approach allows us to identify trends and shifts in perceptions over time, providing valuable insights into the changing landscape of opinions on animal experimentation.

## Materials and methods

Participants

The participants of this study were from four chosen faculties at the EMU in Northern Cyprus: the Faculty of Medicine (Year One to Year Six), the Faculty of Pharmacy (Year One to Year Five), the Faculty of Law (Year One to Year Four), and the Department of Psychology (Faculty of Arts and Sciences) (Year One to Year Four). Faculties of Medicine and Pharmacy were chosen to observe the attitudes toward animal experimentation from a medical and health aspect, whereas Faculties of Law and the Department of Psychology were chosen to observe the attitudes from a psychological and legal aspect. Only students above the age of 18 and currently studying in either of the four faculties mentioned were included in this study. Questionnaires not completely filled out were excluded. A sample size of 215 was calculated using OpenEpi, an online sample size calculator, after inputting the population size of the university (19,730), anticipated frequency (17%) [10], confidence level (95%), and design effect of 1.0. Participants were chosen at random via stratified sampling from student lists provided by their respective faculties or departments. Two out of the 215 participants did not completely fill out the questionnaire and were excluded from this study. The final sample size is 213 (99.07%).

Materials

Data were collected using an open-access, self-administered questionnaire adapted from a study titled “Attitudes Toward Animal Research” conducted by Gallop and Beckstead in 1988 [4]. Permission was granted via email by Gallop. The questionnaire measured the attitudes of participants toward the value of animal research, the availability of alternatives, and their own personal preferences with regard to animal welfare. This questionnaire was revisited by Metzger in 2014 [9]. Comparisons between the three studies will be made to see trends in answers over time. The questionnaire consisted of two sections: Parts A and B. Part A collected sociodemographic information (age, gender, year of study, faculty of study, owning a pet, being a vegetarian, and following animal rights groups on social media). Part B was the questionnaire adapted from Gallop and Beckstead. It contains 14 items. Participants were asked to respond to the items on a five-point scale: 1 = strongly disagree, 2 = disagree, 3 = neutral, 4 = agree, and 5 = strongly agree. The mean values for each question for the groups of interest were measured and compared with each other. Higher values on the items are indicative of attitudes in agreement with each statement, and lower values indicate attitudes that are in disagreement with the items on the questionnaire.

Procedure

The present study, conducted at EMU, Famagusta, Cyprus, is cross-sectional in nature. Stratified random sampling was used to gather the data. The data was analyzed using IBM SPSS Statistics for Windows, Version 25.0 (Released 2017; IBM Corp., Armonk, NY, USA) and was reported with 95% confidence intervals. Microsoft Excel was used for the creation of tables and figures (Microsoft Corporation, Redmond, WA, USA). The Shapiro-Wilk test was conducted to check for normality. The Mann-Whitney U test was chosen due to the non-normal distribution of data, as indicated by the Shapiro-Wilk test, ensuring a robust analysis of demographic and attitudinal variables. Differences were considered statistically significant at p < 0.05. All subjects participated voluntarily. All participants provided written informed consent forms to participate in this study. This study was approved by the EMU Medical School Ethics Committee (protocol number: TPF00-2017-0318). This study was in adherence to the principles of the Declaration of Helsinki.

## Results

The response rate was 213 (99.07%). Eighty-eight (41.31%) of the participants were male, and 125 (58.69%) were female. The mean age was 21.72 ± 2.23. Table 1 summarizes the distribution of participants by faculty and year of study.

**Table 1:** Distribution of participants by faculty and year of study The Faculty of Pharmacy is a five-year program and does not have students in year six. The Faculty of Law, as well as the Department of Psychology, are four-year programs and do not have students in years five or six. The hyphen therefore represents the absence of students in those respective years.

Participants’ corresponding year of study	Number of participants from the Faculty of Medicine (N)	Number of participants from the Faculty of Pharmacy (N)	Number of participants from the Faculty of Law (N)	Number of participants from the Department of Psychology (N)
Year One (N)	7	7	20	26
Year Two (N)	3	6	21	27
Year Three (N)	2	5	23	11
Year Four (N)	2	7	20	15
Year Five (N)	2	8	-	-
Year Six (N)	1	-	-	-
Total number and percentage of participants (N, %)	17 (7.98%)	33 (15.49%)	84 (39.44%)	79 (37.09%)

The diverse distribution of participants across different faculties and years provides a comprehensive representation of attitudes toward animal experimentation across various academic backgrounds.

A total of 161 (75.59%) participants claimed to own a pet. Eighty-eight (41.31%) participants identified themselves as followers of an animal rights organization on social media. Moreover, 175 (82.16%) respondents claimed to not use animals in any form of clothing or decoration, and 167 (78.40%) were against the use of animals for such luxuries. Furthermore, 40 (18.78%) stated that they went fishing and/or hunting as a hobby. When participants were asked about their eating habits, 209 (98.12%) stated that they eat meat, one (0.47%) stated that they did not eat meat due to health problems (hypertension), and three (1.41%) stated that they did not consume meat out of respect for animals.

The most agreed statement in Part B was Q3 (4.42 ± 0.881), “I am very concerned about the pain and suffering of animals,” and the least agreed statement was Q4 (2.17 ± 1.073), “I would rather see humans die or suffer from disease than see animals used in research.” The mean scores of all 14 items in Part B of our study are shown in Table 2. For comparison, the mean scores of the same questionnaire from two other studies are also included: one in 1988 by Gallop and Beckstead [4] and the other in 2014 by Metzger [9].

**Table 2:** Mean scores of Q1-Q14 from 1988, 2014, and the present study Q: question/statement

Statements 1-14 from the questionnaire	1988	2014	Present
Q1: Research on animals has little or no bearing on problems confronting people.	2.05	2.9	2.71
Q2: An intrinsic interest in the animal for its own sake is ample justification for doing animal research.	3.01	2.98	2.49
Q3: I am very concerned about the pain and suffering of animals.	4.02	3.22	4.42
Q4: I would rather see humans die or suffer from disease than see animals used in research.	1.68	2.74	2.17
Q5: Since many important questions cannot be answered by doing experiments on people, we are left with no alternative but to do animal research.	3.57	2.98	2.99
Q6: I have seriously considered becoming a vegetarian in an effort to save animal lives.	1.99	2.79	2.2
Q7: New surgical procedures and experimental drugs should be tested on animals before they are used on people.	3.78	2.98	3.01
Q8: There are plenty of viable alternatives to the use of animals in biomedical and behavioral research.	2.95	3.23	3.48
Q9: Many important biomedical breakthroughs are a consequence of animal research.	3.9	3.2	3.43
Q10: Animals should be granted the same rights as humans.	2.63	2.93	3.41
Q11: Most psychological research done on animals is unnecessary and invalid.	2.57	2.96	3.02
Q12: We need more regulations governing the use of animal research.	3.7	3.2	4.06
Q13: Most laboratory animals are better housed, fed, cared for, and protected from pain and suffering than many humans.	2.81	2.94	2.74
Q14: Animal research cannot be justified and should be stopped.	2.12	2.92	3.14

Compared to earlier years, the present study indicates increased concern for animal welfare, reduced support for extreme views against animal research, and growing acceptance of animal rights. These trends suggest evolving societal attitudes toward animal experimentation and increased consideration for animal rights over time.

Mann-Whitney U tests revealed significant differences for Q4, Q10, and Q14 (p = 0.012, 0.020, and 0.016, respectively) with respect to gender. Regarding these statements, the mean scores of females were 2.88 ± 0.11, 3.61 ± 1.16, and 3.29 ± 1.29, whereas the mean scores of males were 3.19 ± 1.03, 3.14 ± 1.11, and 2.92 ± 1.23, respectively, as seen in Table 3.

**Table 3:** Significant mean score differences between males and females regarding Q4, Q10, and Q14 A p-value <0.05 is deemed to be statistically significant, as indicated by an asterisk (*). Q: question/statement

Select statements from the questionnaire	Female (mean ± SD)	Male (mean ± SD)	p-value
Q4: I would rather see humans die or suffer from disease than see animals used in research.	2.88 ± 0.11	3.19 ± 1.03	0.012*
Q10: Animals should be granted the same rights as humans.	3.61 ± 1.16	3.14 ± 1.11	0.020*
Q14: Animal research cannot be justified and should be stopped.	3.29 ± 1.29	2.92 ± 1.23	0.016*

The table reveals significant gender differences in attitudes toward animal experimentation. Females are more supportive of granting animals the same rights as humans and show stronger agreement that animal research cannot be justified and should be stopped. These differences suggest that female participants generally exhibit greater empathy and support for animal rights.

Table 4 shows that being a pet owner significantly affected the mean scores of Q7 and Q10 (p = 0.046 and p = 0.000, respectively). Thirty-nine (24.22%) of the students who owned pets agreed with Q7: ‘‘New surgical procedures and experimental drugs should be tested on animals before they are used on people,” whereas 27 (51.92%) of the non-pet owners agreed with the same statement. A total of 97 (60.25%) of the students who owned pets agreed with Q10: ‘‘Animals should be granted the same rights as humans,” whereas only 15 (28.85%) of the non-pet owners agreed with the statement.

**Table 4:** Significant differences in agreement regarding Q7 and Q10 between pet owners and non-pet owners A p-value <0.05 is deemed to be statistically significant, as indicated by an asterisk (*). Q: question/statement

Select statements from the questionnaire	Pet owners that agree, N (%)	Non-pet owners that agree, N (%)	p-value
Q7: New surgical procedures and experimental drugs should be tested on animals before they are used on people.	39 (24.22%)	27 (51.92%)	0.046*
Q10: Animals should be granted the same rights as humans.	97 (60.25%)	15 (28.85%)	0.000*

The table indicates significant differences in attitudes between pet owners and non-pet owners regarding animal experimentation. Pet owners are less likely to agree that new surgical procedures and experimental drugs should be tested on animals before humans, and they are more inclined to support granting animals the same rights as humans. These findings suggest that pet ownership may foster empathy toward animals and influence attitudes toward their treatment in research settings.

Table 5 summarizes significant differences that were observed among participants following animal rights organizations on social media, and those that were not, specifically in Q3 (p = 0.043), Q4 (p = 0.028), Q6 (p = 0.001), Q10 (p = 0.003), Q13 (p = 0.010), and Q14 (p = 0.025).

**Table 5:** Significant mean scores among those who do and do not follow animal rights organizations on social media A p-value <0.05 is deemed to be statistically significant, as indicated by an asterisk (*). Q: question/statement

Select statements from the questionnaire	Follows animal rights organizations on social media (mean ± SD)	Does not follow animal rights organizations on social media (mean ± SD)	p-value
Q3: I am very concerned about the pain and suffering of animals.	4.58 ± 0.72	4.34 ± 0.93	0.043*
Q4: I would rather see humans die or suffer from disease than see animals used in research.	2.32 ± 1.03	2.03 ± 1.06	0.028*
Q6: I have seriously considered becoming a vegetarian in an effort to save animal lives.	2.51 ± 1.23	1.97 ± 1.02	0.001*
Q10: Animals should be granted the same rights as humans.	3.70 ± 1.14	3.22 ± 1.15	0.003*
Q13: Most laboratory animals are better housed, fed, cared for, and protected from pain and suffering than many humans.	2.50 ± 1.11	2.90 ± 1.10	0.010*
Q14: Animal research cannot be justified and should be stopped.	3.37 ± 1.37	2.95 ± 1.15	0.025*

The table shows significant differences in attitudes between individuals who follow animal rights organizations on social media and those who do not. Those who follow these organizations are more concerned about animal pain and suffering, more opposed to animal research, more likely to consider vegetarianism, and more supportive of granting animals rights equal to humans. This suggests that engagement with animal rights content on social media influences attitudes toward animal welfare and rights, impacting perceptions of animal experimentation and ethical considerations.

**Figure 1:**
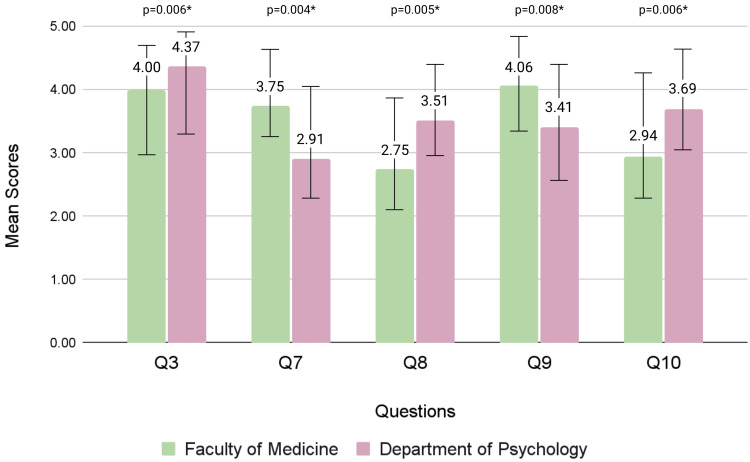
Comparison of significant mean scores between the Faculty of Medicine and the Department of Psychology students A p-value <0.05 is deemed to be statistically significant, as indicated by an asterisk (*).

Having fishing and/or hunting among hobbies had a significant effect on the means of Q7 (p = 0.017) and Q9 (p = 0.007). Being against the use of animals for clothing or decoration purposes significantly impacted 11 of the 14 items in Part B, with the exceptions being Q1 (p = 0.180), Q6 (p = 0.117), and Q8 (p = 0.142). The mean scores of the Faculties of Medicine, Pharmacy, Law, and Psychology were compared with each other to assess the impact of the field of study on attitudes toward animal experimentation. Significant differences were observed among mean scores between medical and psychology students for Q3 (p = 0.006), Q7 (p = 0.004), Q8 (p = 0.005), Q9 (p = 0.008), and Q10 (p = 0.006), as summarized in Figure 1.

**Figure 2:**
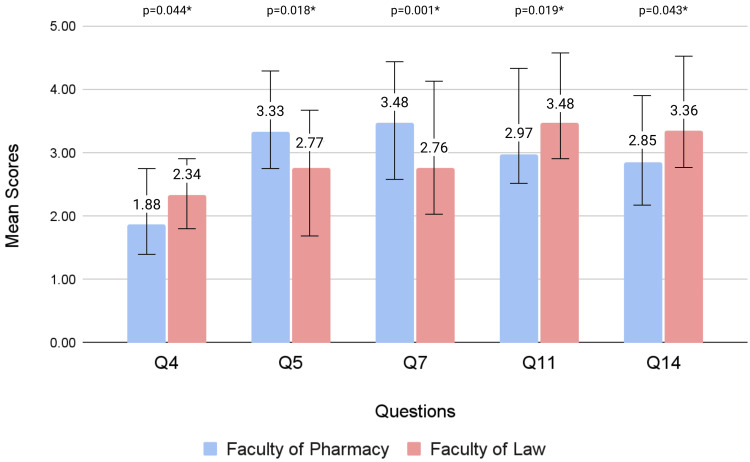
Comparison of significant mean scores between the Faculty of Pharmacy and the Faculty of Law students A p-value <0.05 is deemed to be statistically significant, as indicated by an asterisk (*).

Significant differences were observed among mean scores between pharmacy and law students for Q4 (p = 0.044), Q5 (p = 0.018), Q7 (p = 0.001), Q11 (p = 0.019), and Q14 (0.043), as summarized in Figure 2.

**Figure 3:**
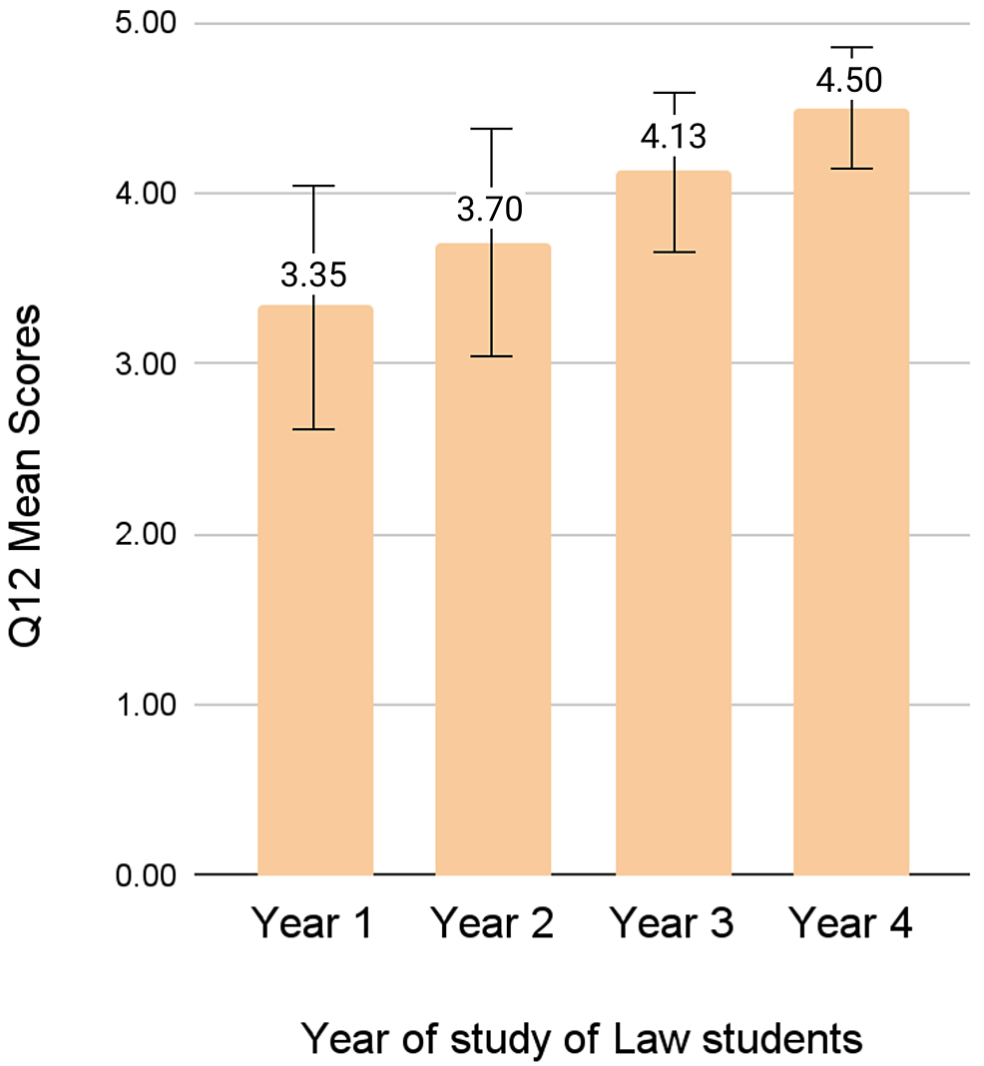
Comparison of Q12 mean scores among law students from Year One to Year Four of education

When health-related faculties, Medicine and Pharmacy, were compared to each other, significant differences in mean scores were found in Q3 (p = 0.001) and Q9 (p = 0.040). When non-health-related faculties, Law and Psychology, were compared with each other, a significant difference was found in only Q11 (p = 0.000). No significant differences were detected among mean scores between first-year and final-year students in any of the faculties. However, regarding Q12 (“We need more regulations governing the use of animal research”), we can observe a gradual increase in the mean scores among law students as they progress from their first to fourth year of education (first year: 3.35 ± 1.39, second year: 3.70 ± 1.34, third year: 4.13 ± 0.97, and fourth year: 4.50 ± 0.76), as can be appreciated in Figure 3.

## Discussion

The participants of this study seemed to show less acceptance of animal experimentation and more acceptance of its alternatives when compared to similar studies using the same questionnaire. This is likely due to the development of technology. With each passing day, more sophisticated models of the human body are being produced, essentially eliminating the need for animal research in certain fields. This finding aligns with Knight et al., who reported that the general public tends to be less supportive of animal experimentation than scientists [1].

The concept of animal rights has been evolving over the years as a result of the successful infiltration of animal rights organizations on social media [5]. These organizations have been successful in raising public awareness regarding animal experimentation. Ormandy and Schuppli note the significant impact that animal rights organizations and social media have on shaping public perceptions and attitudes toward animal rights [5]. This explains why, over time, people have started to believe more and more that animals should be granted the same rights as human beings, especially those who follow animal rights associations on social media. Participants following such organizations on social media were more inclined to consider becoming vegetarian in an effort to save animal lives. Social media has likely influenced this inclination.

Significant differences in mean scores were observed between participants majoring in non-health-related fields and those in health-related fields. Those studying medicine or pharmacy were more likely to have a positive attitude toward animal experimentation, whereas those studying psychology or law were more likely to have a negative attitude toward animal experimentation. This is likely because the students in the health-related faculties are both more aware and more educated regarding this topic as a result of their academic curriculum. They are also aware that, with today’s technology, it is not possible to completely replace animal experimentation with its alternatives.

The findings of this study also revealed significant differences in attitudes according to the participant’s gender. Female participants were more concerned about the animals used in the experimentation in comparison to their male counterparts. Graça et al. support this finding, noting that women typically express greater concern for animal welfare than men [11]. Females argued that animal research cannot be justified and should be stopped and that most psychological research done on animals is unnecessary and invalid. Female participants agreed more strongly that animals should be granted the same rights as humans. Male participants, however, were more supportive of animal research and agreed that new surgical procedures and experimental drugs should be tested on animals first before being used on people. Traditional gender expectations still persist today, in which women are expected by society to be more gentle, sensitive, and compassionate as opposed to men. This difference likely arises from sociocultural differences between men and women. Men are expected to be protective of those around them, and this concept is proven in their support for animal experimentation and their urge to save human lives as opposed to animal lives.

Final-year law students, in comparison to first-year students, were more of the opinion that laws and regulations governing the use of animals in research are necessary. This is likely because final-year law students are in their fourth year of education and have a broader and more sophisticated understanding of the necessity of laws and regulations. First-year students, on the other hand, are fresh out of high school and may not be as immersed in this subject. Similarly, Machado et al. found that senior veterinary students were more favorable to animal experimentation and supportive of the regulations surrounding animal use in research and animal welfare, whereas junior veterinary students were more opposed to animal experimentation as a whole [12].

Significant differences were detected between those who were against the use of animals for clothing or decoration purposes and those who were not against it. Those against the use of animals in such a manner were more of the opinion that we need more regulations governing the use of animals in research. Not wanting to use an animal for cosmetic purposes tells us that participants respect the animal and its body, even after death. The use of animals for decoration is not a necessity and cannot be justified, as there is no direct benefit to humans.

It is clear that pet ownership has an influence on opinions toward the use of animals in research. Pet owners were of the idea that animals should be granted the same rights as human beings, whereas those who did not have pets strongly believed that new surgical procedures and experimental drugs should be tested on animals before they are used on humans. Serpell also found that pet ownership is associated with greater empathy toward animals and more positive attitudes toward their welfare [13]. The literature further tells us that having pets while growing up leads to the development of a positive and caring attitude toward animals later on in life. According to the “contact theory,” contact with nonhuman animals leads to mutual understanding and decreased prejudice between the person and the nonhuman animal [14]. Positive encounters with animals generally lead to positive attitudes toward animals, whereas negative experiences with animals may make a person more supportive of the use of animals in experimentation and research [13]. Most pet owners consider their pets as a part of their family, which makes sense that they would not support the use of animals in research. We believe that a familial bond is formed between a person and their pet and that this is the reason why pet owners are more protective and caring of animals.

Interestingly, those who go hunting or fishing as a hobby agree that new surgical procedures and experimental drugs should be tested on animals first before they are used on humans. This makes sense because if a person enjoys hunting down and killing animals for sport, then they would have no objection to animals being experimented on. We believe that participants who have such hobbies are accustomed to the pain and suffering of animals because they have seen it many times before and may think that there is nothing unusual about it. Some contend that hunting requires the hunter to empathize with the animal, potentially fostering heightened sympathy and a broader concern for animals, a viewpoint that contrasts with the findings of the current study [15].

Limitations

The current study was conducted exclusively among four faculties at EMU in Northern Cyprus. Including a broader range of universities and faculties would enhance the generalizability of our findings to the entire country or region. Furthermore, due to the limited number of vegetarians among our participants, comparisons between vegetarians and nonvegetarians should be interpreted cautiously. Future studies should aim to recruit a more representative sample and increase the number of vegetarians to better understand their attitudes toward animal experimentation. Lastly, our study exclusively examined the attitudes of university students toward animal experimentation, thereby limiting the generalizability of the findings to educators at the university. Exploring the perspectives of teachers, professors, and lecturers would offer insights into the impact of professional roles on attitudes toward animal research. These steps are crucial for advancing our understanding of societal attitudes and perceptions regarding animal experimentation in broader contexts.

## Conclusions

The cross-sectional study at EMU reveals a complex and evolving landscape of attitudes toward animal experimentation among university students. While there is a significant concern for the welfare of animals used in research, there is also a recognition of the necessity of such experiments in advancing medical and scientific knowledge. Factors such as gender, pet ownership, and field of study play crucial roles in shaping these attitudes. The findings underscore the importance of continued education on the humane treatment of research animals and the ethical considerations involved in animal experimentation. To promote ethical research practices, we recommend implementing mandatory courses on animal research ethics across disciplines, emphasizing humane treatment, and exploring alternative methods. As technology advances and alternative methods become more viable, it is essential to balance scientific progress with animal welfare. This study contributes to the broader discourse on animal research ethics by offering insights into evolving societal perspectives and emphasizing the need for ongoing dialogue and research to ensure that scientific progress aligns with ethical standards. Future research should expand to include a broader demographic to better understand these attitudes and track changes over time, ensuring that animal research practices are both ethically sound and scientifically justified.
